# Impact of drought stress on vitamin C and anthocyanin content in cultivated lettuces (*Lactuca sativa* L.) and wild relatives (*Lactuca* spp.)

**DOI:** 10.3389/fpls.2024.1369658

**Published:** 2024-03-15

**Authors:** Inés Medina-Lozano, Juan Ramón Bertolín, Aurora Díaz

**Affiliations:** ^1^ Department of Plant Science, Agrifood Research and Technology Centre of Aragon (CITA), Zaragoza, Spain; ^2^ AgriFood Institute of Aragon – IA2, CITA-Universidad de Zaragoza, Zaragoza, Spain; ^3^ Department of Animal Science, Agrifood Research and Technology Centre of Aragon (CITA), Zaragoza, Spain

**Keywords:** lettuce, crop wild relatives, antioxidants, ascorbic acid, UPLC-UV, abiotic stress, water deficit, resilience

## Abstract

**Introduction:**

Lettuce production and quality could be seriously affected by the increasingly limited water resources.

**Methods:**

The effect of drought on the content of two antioxidant compounds, vitamin C and anthocyanins, in five cultivated lettuces and two wild relatives was assessed for 2 years.

**Results and discusion:**

In leaf samples, *Lactuca* wild species generally had a higher content of total vitamin C than the cultivated lettuces. In contrast, the commercial varieties usually contained more total anthocyanins than the wild species. Total vitamin C decreased with the drought stress in all accessions, commercial varieties, and lettuce wild relatives, with this tendency being consistent and reproducible across the 2 years. These differences were significant in the case of the green commercial varieties ‘Winter Crop’ (in 2020/2021) and ’Dolomiti G12’ (in 2021/2022) and very significant in the red commercial variety ’Red Sails’ (in 2020/2021). However, the only group in which the effect of drought was either significant or very significant in both years was the wild species, *Lactuca homblei* and *Lactuca dregeana*, and in the latter also in both tissues (leaf and stem) analyzed. Water stress resulted in an increase of the total anthocyanin content in the leaves from all the accessions, both red commercial varieties and wild relatives, in both years. The most significant enrichment and the only one being either significant or very significant in both years was observed in one of the wild relatives assayed (*L. homblei*). Stems (*L. dregeana*) contained more anthocyanins than leaves under control conditions, and it was exactly the opposite under drought. Changes in anthocyanins in the two tissues in response to drought stress were in opposite directions, increasing in leaves and decreasing in stems. This could suggest a translocation of anthocyanins as a first quick mechanism to cope with a severe lack of water. In conclusion, anthocyanins (unlike vitamin C) could play a role in the mechanisms deployed by the plant to tolerate drought stress. The wild species with a robust significant enrichment in anthocyanins as a response to drought (*L. homblei*) is a promising plant material to breed more resilient lettuces.

## 1 Introduction

At present, there is a consensus within the scientific community about the planet being immersed in a climate change scenario. There are many consequences of global warming caused or, at least, accelerated by human activity. Among them, one of the most worrying for agriculture consists of the more frequent, longer, and more severe droughts, especially in the Mediterranean Basin (IPCC 2021). This will likely result in economic costs due to crop yield losses, which, in turn, could lead to an increase in food prices. Because of their sessile nature, plants have deployed sophisticated and interconnected mechanisms to survive a wide range of environmental threats (Catalá et al., 2012), and the challenge for scientists lies in uncovering and enhancing them.

Lettuce (*Lactuca sativa* L.) is one of the major leafy vegetable crops worldwide (FAOSTAT, 2021). Over the years, lettuce varieties have been improved through breeding, mainly to increase production and to introduce resistance to diseases. Actually, most of the QTL (quantitative trait loci) or genes mapped in the lettuce genome are responsible for resistance to diseases and pests (Simko et al., 2021). This has resulted in commercial varieties that are, in most cases, nutritionally poor, especially when compared to other leafy vegetables, like chard or spinach (Haytowitz et al., 2015), and not very resilient due to its low tolerance to some adverse environmental conditions, such as drought. In general, crop wild relatives (CWR) have been widely used to increase the crop resistance to biotic stresses (Dempewolf et al., 2017). In contrast, CWR have barely been considered to improve the quality of the crops, even if sometimes they can be richer than the commercial varieties in certain nutrients, like vitamin C, as it happens to be the case in some *Lactuca* spp (van Treuren et al., 2018; Medina-Lozano et al., 2021). In the case of lettuce, CWR have not been either exploited to enhance the tolerance to abiotic stresses, like drought.

Water deficit is known to cause the accumulation of antioxidants in some crops, though in lettuce, this has barely been studied yet. That is why this research is aimed at assessing the effect of drought stress on the content of two phytochemicals with high antioxidant capacity, vitamin C and anthocyanins, in both cultivated lettuces and wild relatives. Both types of compounds have health-promoting properties mainly due to their antioxidant activity (Cásedas et al., 2020; Xu et al., 2022) and have been reported to participate in the plant defense against biotic stresses and in mechanisms of tolerance to abiotic stresses (Gould, 2004; Locato et al., 2013 and references herein). Vitamin C or total ascorbic acid (TAA) is made up of ascorbic acid (AA) and its oxidation product, dehydroascorbic acid (DHAA) (Medina-Lozano et al., 2020). Vitamin C is an indicator of the quality of many fruits and vegetables that must be incorporated in the diet as it is an essential micronutrient for humans (Carr and Frei, 1999). Anthocyanins are phenolic compounds, specifically flavonoids (Medina-Lozano and Díaz, 2021), responsible for the red color of the leaves of some lettuce varieties. In lettuce, most research on anthocyanins have focused on studying the effect of environmental factors, such as temperature, photoperiod, and wavelength of the artificial light supplied. The content of anthocyanins seems to raise generally in plants when they are grown at low temperatures and with blue light (Li and Ahammed, 2023), which has also been confirmed in lettuce (Chon et al., 2012). Regardless of the environmental effects, the contents of vitamin C and anthocyanins have an important genetic component, with great differences between accessions. Lettuce wild relatives have been described as the richest in vitamin C, followed by the traditional varieties and then the commercial varieties, with exactly the inverse order in the case of the anthocyanins (Medina-Lozano et al., 2021). In other crops, like vine, red grapes accumulate anthocyanins under drought conditions due to a greater and earlier expression of genes of the biosynthesis pathway (Castellarin et al., 2007a; Castellarin et al., 2007b). In fact, in lettuce, the addition of some elicitors, which are phytohormones that participate in a multitude of mechanisms of resistance and tolerance to biotic and abiotic stresses, respectively, increases the content of certain compounds, such as vitamin C (in response to jasmonic acid and arachidonic acid) and some polyphenols (in response to abscisic acid, jasmonic acid, and arachidonic acid) (Złotek et al., 2014). However, a deeper and more thorough investigation on the subject is needed as a very recent study in lettuce landraces shows differing results, with a contrary effect of drought stress on vitamin C (decrease) and phenolic compounds (increase), in response to the water stress (Ćavar Zeljković et al., 2023).

Our hypothesis is that the plant could increase the amount of certain compounds with antioxidant power when subject to stress, in this case, drought, as a tolerance mechanism. That is why in this study, we are interested in assessing the changes in some antioxidant compounds (i.e., vitamin C and anthocyanins) in lettuce commercial varieties and some wild relatives under drought stress. Recently, the changes in the amounts of vitamins and phenolic compounds (among other phytochemicals) in response to saline and water stress have been assessed in six cultivated lettuce accessions (Ćavar Zeljković et al., 2023). We believe that it is important to also include CWR in these types of studies because they are expected to show a higher tolerance to adverse environmental conditions. With domestication, crops have become dependent on guaranteed inputs (water, fertilizers, phytosanitary products, etc.), in contrast to the CWR, which will have to cope with the adverse climatic and phytosanitary conditions by evolving endogenous strategies of tolerance and defense. We believe that the enhancement of lettuce resilience could be of great interest as a basis for undertaking future breeding programs.

## 2 Materials and methods

### 2.1 Plant material and water stress assays

**Figure 1:**
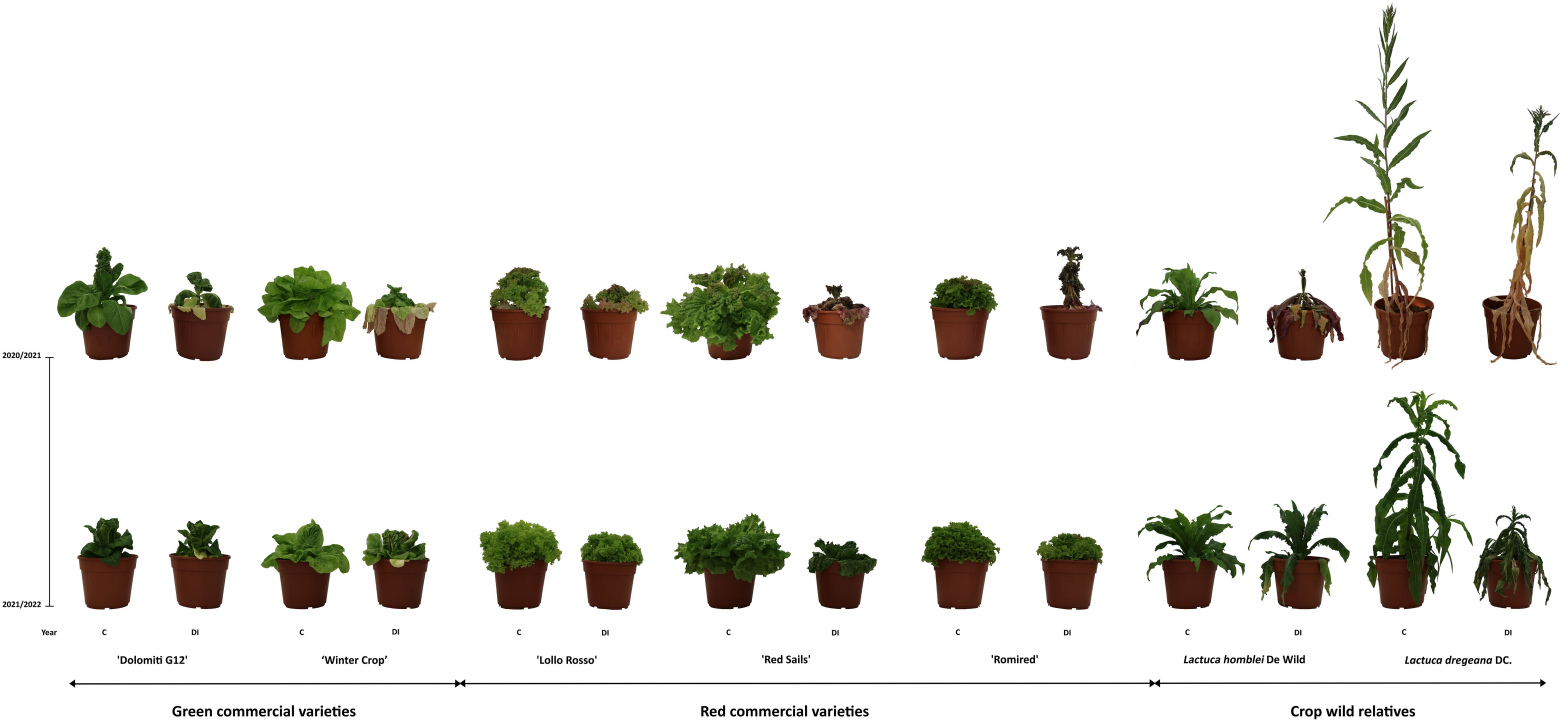
Commercial (green and red) lettuce varieties, as well as wild relatives (*Lactuca* spp.), under control (C) and drought stress (DI) experiments carried out during 2020/2021 and 2021/2022.

Seven *Lactuca* accessions were included in this study (**Table 1** and **Figure 1**): five lettuce commercial varieties, two green (‘Dolomiti G12’ and ‘Winter Crop’) and three red (‘Lollo Rosso’, ‘Red Sails’, and ‘Romired’), and two wild relatives (*Lactuca dregeana* DC. and *Lactuca homblei* De Wild). Plants were cultivated for 2 weeks in a growing chamber at 25°C with an average relative humidity of 50% and a short-day photoperiod (10 h light/14 h darkness). Then, they were transplanted to pots (30 × 25 cm and 11.7 L volume) containing a mix of black and blonde peat (1:1) supplemented with fertilizer in a greenhouse at Agrifood Research and Technology Centre of Aragon (CITA, Zaragoza, Spain). Maximum temperature was set at 35°C, relative humidity was set at 40%, and no supplemental lighting was supplied to avoid the enhancement of anthocyanin synthesis (natural photoperiod from December to March).

**Table 1:** Description of the plant material used in the water stress study, commercial lettuce varieties, and wild relatives (*Lactuca* spp.).

Name	Species	Group	Leaf color	Source^b^	Accession number
‘Dolomiti G12’	*Lactuca sativa* L.	Commercial variety	Green	Ramiro Arnedo Semillas S.A.	–
‘Winter Crop’	*Lactuca sativa* L.	Commercial variety	Green	CGN	CGN05853
‘Lollo Rosso’	*Lactuca sativa* L.	Commercial variety	Red^a^	CGN	CGN09385
‘Red Sails’	*Lactuca sativa* L.	Commercial variety	Red^a^	CGN	CGN19014
‘Romired’	*Lactuca sativa* L.	Commercial variety	Red^a^	CGN	CGN24713
*Lactuca dregeana*	*Lactuca dregeana* DC.	Wild crop relative	Dark green (red stems)	BGHZ	BGHZ3670
*Lactuca homblei*	*Lactuca homblei* De Wild	Wild crop relative	Green (red nerves)	BGHZ/CGN	BGHZ5322/CGN11322

a Semi-red under our experimental conditions.

b BGHZ: Vegetable Germplasm Bank of Zaragoza (Spain); CGN: Centre for Genetic Resources (Wageningen, Netherlands).

-, not assigned.

Water stress experiments were repeated two consecutive years, in winters 2020/2021 and 2021/2022. They consisted of two extreme irrigation regimes, control (C) or full irrigation (week 1: 1,350 mL, weeks 2–3: 2,100 mL/each) and deficit irrigation (DI) (weeks 1–3: 0 mL) scheduled 3 weeks before harvesting, which happened to be approximately 3 months after transplanting. More moderate deficit irrigation regimes tested in preliminary experiments carried out in winter 2018/2019 (DI-1: 700 mL and DI-2: 500 mL during only the 2 weeks before harvesting) did not cause any perceivable stress in the plants (data not shown). In both years (2020/2021 and 2021/2022), three plants per accession were assayed in a complete randomized block design, with three biological replicates per block, up to a total of 42 plants.

Two leaves representing the whole plant (i.e., inner and outer leaves in plants that grow forming rosettes or heads), as well as parts of the stem in the case of *L. dregeana* (it grows as a bush), were harvested (**Figure 1**). All the plant material was immediately frozen using liquid nitrogen and then preserved at −80°C until their lyophilization, as described previously (Medina-Lozano et al., 2020).

### 2.2 Phytochemical quantification

#### 2.2.1 Extraction and chromatographic determination of vitamin C

As previously described (Medina-Lozano et al., 2020), vitamin C or TAA was quantified as the sum of AA and DHAA in 50 mg of finely powdered lyophilized samples (**Table 1**). Briefly, AA was extracted with a solution of 8% acetic acid (v/v), 1% MPA (w/v) (both from Sigma-Aldrich, Madrid, Spain), and 1 mM EDTA (Panreac, Barcelona, Spain). After vortexing the samples for 5 s and shaking them for 10 min at 2,000 rpm, they were sonicated for 10 min at room temperature and centrifuged at 4,000 × *g* for 10 min at 4°C. Finally, Extract 1 (E1) was obtained by filtering the supernatant through a 0.22-µm regenerated cellulose filter (Agilent, CA, United States). A 200-µL aliquot of that extract was used to transform DHAA into AA with 200 µL of reducing solution (40 mM DTT with 0.5 M Tris, pH 9.0) (Roche, Madrid, Spain) during 30 min at room temperature in darkness. The reaction was stopped and made stable at acidic pH by adding 200 µL of 0.4 M sulfuric acid (Extract 2, E2).

AA and TAA were quantified by preparing dilutions of E1 and E2, respectively, with ultrapure water (1:4 v:v) and using a liquid chromatographer UPLC H-Class with an HSS T3 column (150 mm × 2.1 mm × 1.8 μm), and an Photodiode Array PDA eλ Detector, controlled by Empower 3 software (Acquity, Waters, Mildford, MA, USA). TAA was composed of AA coming from both sources in E2 (AA present in the extract and AA coming from the reduction of DHAA). DHAA content was easily calculated subtracting AA from TAA. This indirect approach is required because DHAA has a low absorptivity in the UV range of the spectrum, which makes its quantification difficult. The flow rate consisted of 0.3 mL min^−1^ of 2% methanol (ChemLab, Zedelgem, Belgium) and 98% ultrapure water, pH 2.0, acidified with formic acid (Supelco-Sigma-Aldrich) in isocratic mode. A volume of 5 µL of each extract kept at 5°C was injected and run through the column at 30°C for 3 min. AA was identified and quantified using an external calibration curve built with the commercial standard L-ascorbic acid (≥99.9% purity, Sigma-Aldrich) and the photodiode array detector at 245 nm. The analytical method is described in more detail in Medina-Lozano et al. (2020).

#### 2.2.2 Extraction and chromatographic determination of anthocyanins

The extraction of anthocyanins was carried out with samples coming from all the accessions described in **Table 1** (green-leaf varieties included) following the method described by Assefa et al. (2019) and adapted by Medina-Lozano et al. (2021) in an environment with low intensity light to prevent anthocyanin degradation. Succinctly, a double extraction with 40 mg of fine powder of lyophilized plant material and 5 mL of methanol:ultrapure water:formic acid solution (50:44:6 v:v:v) was carried out. After vortexing the samples for 5 s and shaking them for 20 min at 2,000 rpm, they were sonicated for 20 min at room temperature and centrifuged at 4,000 × *g* for 10 min at 4°C. Finally, the mixed supernatants from both extractions were passed through a 0.22-µm polytetrafluoroethylene (PTFE) filter (Agilent).

For the chromatographic determination of the anthocyanins, the method described by Fernández-Barbero et al. (2019) with slight modifications (Medina-Lozano et al., 2021) was followed. In brief, the separation of the specific anthocyanins was carried out by liquid chromatography in an Acquity UPLC BEH C18 column (50 mm × 2.1 mm × 1.7 µm, Waters). Methanol (A) and ultrapure water pH 2.0 acidified with formic acid (C) were used as mobile phases at a flow rate of 0.3 mL min^−1^ in gradient mode of A and C (**Supplementary Table S1**). A volume of 3 µL of each extract kept at 10°C was injected and run through the column at 30°C for 20 min, with chromatograms acquired at 520 nm.

The patterns obtained in a previous work in which these *Lactuca* accessions together with many others were characterized in terms of anthocyanin content (Medina-Lozano et al., 2021) allowed us to identify the same three anthocyanins found before: cyanidin 3-*O*-(6’-*O*-malonylglucoside), cyanidin 3-(6’’-acetylglucoside), and peonidin 3-*O*-glucoside. Their quantification was performed building the respective calibration curves, with ≥96% purity Kuromanin chloride or cyanidin 3-*O*-glucoside chloride (from 0.1 to 50 µg mL^-1^) for the two cyanidin derivates and with ≥95% purity peonidin 3-*O*-glucoside chloride (0.01 to 1 µg mL^-1^) for the peonidin-type anthocyanin, both purchased from Extrasynthese (Genay, France).

### 2.3 Method validation

The chromatographic methods were validated in both cases, vitamin C and anthocyanin determination, by calculating different analytical parameters [selectivity, sensitivity, limit of detection (LOD), limit of quantification (LOQ), linearity of the calibration curve tested by the coefficient of determination (*R* ^2^), repeatability and intermediate precision expressed as coefficients of variation (CV, %), and recovery (Rec, %) as previously described (Bertolín et al., 2018; Medina-Lozano et al., 2021)].

### 2.4 Data analysis

Characteristics in terms of the content of vitamin C (TAA, AA, and DHAA) and of both specific [cyanidin 3-*O*-(6’-*O*-malonylglucoside), cyanidin 3-(6’’-acetylglucoside), and peonidin 3-*O*-glucoside] and total anthocyanins by treatment (C *vs*. DI) within accessions were assessed by descriptive statistics in 2020/2021 and 2021/2022 (**Table 2**; **Supplementary Tables S2**, **S3**). One-way analysis of variance (ANOVA) was also employed to compare the means of all the accessions within each irrigation regime (C and DI) for every year (2020/2021 and 2021/2022). The means of all pairs of accessions were compared using the unpaired *t*-test. Non-normally distributed data were transformed {[TAA]^2^ for DI in 2021/2022 and ln (*anthocyanins*) for C in 2020/2021 and 2021/2022, and for DI in 2021/2022}. Equal variances could be assumed in all cases. The number of biological replicates was 3 (*n* = 3).

**Table 2:** Total ascorbic acid (TAA) and total anthocyanin content in commercial lettuce varieties and some wild relatives (*Lactuca* spp.) under control (C) and drought stress (DI) conditions in a 2-year experiment.

			2020/2021				2021/2022			
			TAA (mg 100 g^−1^ DW)		Anthocyanins (mg 100 g^−1^ DW)		TAA (mg 100 g^−1^ DW)		Anthocyanins (mg 100 g^−1^ DW)	
Name	Group	Tissue	C	DI	C	DI	C	DI	C	DI
‘Dolomiti G12’	Green commercial variety	Leaf	251.73 ± 39.06^cd^	195.60 ± 11.48^ab^	ND	ND	245.02 ± 7.42^cd^	231.17 ± 3.65^ab^	ND	ND
‘Winter Crop’		Leaf	322.58 ± 50.82^b^	188.79 ± 17.72 ^ab^	ND	ND	248.03 ± 21.7^b^	239.73 ± 26.89^ab^	ND	ND
‘Lollo Rosso’	Red commercial variety	Leaf	225.80 ± 21.35^d^	206.65 ± 12.01^a^	70.27 ± 43.44^ab^	74.41 ± 6.17^cd^	221.14 ± 11.96^d^	204.49 ± 18.07^a^	3.01 ± 1.94^b^	4.32 ± 2.88^c^
‘Red Sails’		Leaf	298.34 ± 11.39^bc^	195.32 ± 33.65^ab^	260.8 ± 179.35^a^	356.9 ± 63.9^a^	302.43 ± 39.28^bc^	251.5 ± 24.22^ab^	15.21 ± 6.96^a^	26.55 ± 8.75^a^
‘Romired’		Leaf	253.95 ± 65.63^cd^	213.95 ± 7.31^a^	147.02 ± 68.19^a^	180.09 ± 29.63^bc^	237.04 ± 12.06^cd^	206.14 ± 47.77^a^	31.67 ± 5.54^a^	35.57 ± 12.6^a^
*L. homblei*	Wild crop relative	Leaf	453.85 ± 25.56^a^	224.09 ± 59.18^a^	32.41 ± 16.59^bc^	232.29 ± 122.05^b^	345.53 ± 20.25^a^	285.14 ± 20.94^a^	3.8 ± 0.52^b^	7.02 ± 0.55^bc^
*L. dregeana*		Leaf	355.70 ± 8.55^b^	124.87 ± 65.72^b^	10.7 ± 7.98^c^	21.23 ± 7.59^d^	300.99 ± 13.88^b^	187.01 ± 28.76^b^	4.06 ± 3.24^b^	11.22 ± 3.44^b^
		Stem	72.84 ± 6.13	33.36 ± 5.24	16.97 ± 8.07	9.82 ± 3.86	126.83 ± 11.79	62.63 ± 24.74	12.18 ± 4.25	7.93 ± 2.73

Different letters indicate significant differences among accessions within each treatment (C, DI) at p < 0.05 for leaf samples.

ND: not detected.

The effect of the irrigation regimes assayed (C *vs*. DI) on TAA content in each of the five commercial lettuce varieties and the two wild relatives (*Lactuca* spp.) was studied by unpaired *t*-test (*α* = 0.05) in 2020/2021 and 2021/2022. For the content of anthocyanins, only the three red-leaf commercial varieties plus the two wild species were included as anthocyanins were absent in the two green-leaf commercial varieties, as expected. For the branching wild species (*L. dregeana*), the analyses were carried out with the leaves and also with the stems. Data were transformed to achieve normality only in two cases (1/[TAA]^10^ in 2020/2021 and [TAA]^10^ in 2021/2022) for the red-leaf commercial variety ‘Romired’. Equal variances could always be assumed (homoscedasticity was fulfilled).

All the described statistical analyses were conducted with the software JMP v5.1.2 for Windows (SAS Institute Inc., Cary, NC).

## 3 Results

### 3.1 Quantification of antioxidant compounds and their changes in response to drought stress

#### 3.1.1 Total vitamin C

**Figure 2:**
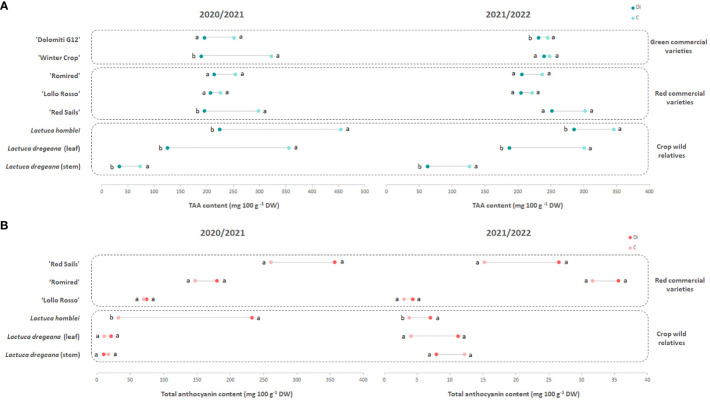
Dumbbell plots of the **(A)** TAA (total ascorbic acid) and **(B)** total anthocyanin contents of green and red commercial lettuce varieties and wild relatives (*Lactuca* spp.) under different irrigation regimes, C (for control) or full irrigation (week 1: 1,350 mL, weeks 2–3: 2,100 mL/each) and DI (for deficit irrigation) (weeks 1–3: 0 mL) in 2 consecutive years (2020/2021 and 2021/2022). Different letters show significant differences (*p* < 0.05) between the two irrigation regimes assayed each year.

In 2020/2021, the mean TAA content in leaves in C conditions ranged between 225.80 and 453.85 mg 100 g^−1^ DW (**Table 2**; **Figure 2A**), in a red-leaf commercial variety (‘Lollo Rosso’) and in a wild relative (*L. homblei*), respectively. In 2021/2022, the results were very similar, with slight movements in the ranking positions with, again, ‘Lollo Rosso’ being the poorest accession in TAA content in leaves (221.14 mg 100 g^−1^ DW) and *L. homblei* being the richest (345.53 mg 100 g^−1^ DW) (**Table 2**; **Figure 2A**). In both years, the differences among accessions were highly significant (*p* = 0.00005 and *p* = 0.00003, respectively).

Under drought stress (DI), the results were again reproducible between years as the accession with the lowest TAA content in leaves was *L. dregeana* (124.87 mg 100 g^−1^ DW in 2020/2021 and 187.01 mg 100 g^−1^ DW in 2021/2022) and the one with the highest was *L. homblei* (224.09 mg 100 g^−1^ DW in 2020/2021 and 285.14 mg 100 g^−1^ DW in 2021/2022), the latter being also the richest under C conditions (**Table 2**; **Figure 2A**), as mentioned above. Even though differences were not significant (*p* = 0.1275) and very significant (*p* < 0.01), respectively, in both years, *t*-test rendered groups with significant differences when paired means were compared (**Table 2**).

In all cases, in both years and irrigation regimes, the samples with the lowest TAA concentrations were the stem tissues collected from the wild species *L. dregeana* (**Table 2**; **Figure 2A**), which was the only plant with bushy growing habits among the studied (**Figure 1**). Those values were also lower than the TAA content of the leaf samples from the same accession, in total agreement with what has been described before (Medina-Lozano et al., 2021). Considering all data (from both years and the two irrigation regimes) within each accession, AA was the most abundant form of vitamin C in most cases, only in ‘Lollo Rosso’ and ‘Dolomiti G12’ was DHAA either always or in half of the cases the predominant compound, respectively (**Supplementary Tables S2**, **S3**).

The accessions under study were chosen to gather a wide range of variability, from those with a high content in TAA, that is, the CWR (*L. dregeana* and *L. homblei*), to those poor in TAA, that is, the cultivated varieties, both green (‘Dolomiti G12’ and ‘Winter Crop’) and red (‘Lollo Rosso’, ‘Red Sails’ and ‘Romired’), according to the classification from Medina-Lozano et al. (2021).

TAA content decreased with the drought stress (DI) in all accessions, commercial varieties (both green and red), and CWR, with that tendency being consistent and reproducible across the 2 years in which the experiments were carried out (**Table 2**). In most cases, the drop in TAA quantity could be observed in both forms of vitamin C, AA and DHAA, and only in a few cases did the reduction happen in only one of them, mainly in AA (**Supplementary Tables S2**, **S3**). These differences in TAA content in leaves between treatments (C *vs*. DI) were significant in the case of the green commercial varieties ‘Winter Crop’ (in 2020/2021; *p* = 0.013) and ‘Dolomiti G12’ (in 2021/2022; *p* = 0.044) and very significant in the red commercial variety ‘Red Sails’ (in 2020/2021; *p* = 0.007) and the two CWR, *L. homblei* (in 2020/2021, *p* = 0.004 and in 2021/2022, *p* = 0.023) and *L. dregeana* (in both 2020/2021 and 2021/2022, *p* = 0.004). In *L. dregeana* stems, the differences were also statistically significant (in 2020/2021, *p* = 0.001 and in 2021/2022, *p* = 0.046) (**Table 3**; **Figure 2A**).

**Table 3:** Effect of drought stress conditions assayed on the total ascorbic acid (TAA) and total anthocyanin contents in commercial lettuce varieties and some wild relatives (*Lactuca* spp.) in a 2-year experiment.

			TAA				Anthocyanins			
			2020/2021		2021/2022		2020/2021		2021/2022	
Name	Group	Tissue	t Ratio	Prob > |t|^a^	t Ratio	Prob > |t|^a^	t Ratio	Prob > |t|^a^	t Ratio	Prob > |t|^a^
‘Dolomiti G12’	Green commercial variety	Leaf	2.388	0.075	2.900	0.044	–	–	–	–
‘Winter Crop’		Leaf	4.305	0.013	0.416	0.699	–	–	–	–
‘Lollo Rosso’	Red commercial variety	Leaf	1.354	0.247	1.331	0.254	−0.163	0.878	−0.650	0.551
‘Red Sails’		Leaf	5.023	0.007	1.912	0.129	−0.874	0.431	−1.756	0.154
‘Romired’		Leaf	−1.046^b^	0.355^b^	1.182^b^	0.303^b^	−0.770	0.484	−0.491	0.649
*L. homblei*	Wild crop relative	Leaf	6.173	0.004	3.591	0.023	−2.811	0.048	−7.345	0.002
*L. dregeana*		Leaf	6.033	0.004	6.182	0.004	−1.655	0.173	−2.625	0.059
		Stem	8.480	0.001	3.299	0.046	1.384	0.239	1.406	0.254

a α = 0.05 used to test significant differences in TAA and total anthocyanin contents between control and deficit irrigation conditions.

b Data transformed (1/[TAA]^10^ in 2020/2021 and [TAA]^10^ in 2021/2022) to achieve normality.

-, not analysed because no anthocyanins were detected.

#### 3.1.2 Total anthocyanins

Anthocyanins were quantified in all the accessions (**Table 1**; **Figure 1**), the two green commercial varieties included, which served as negative controls of the method because, as expected, they did not contain any at all (**Table 2**; **Supplementary Tables S2**, **S3**).

In 2020/2021, the average total anthocyanin content in leaves in C conditions ranged between 10.70 and 260.80 mg 100 g^−1^ DW (**Table 2**; **Figure 2B**). The accession with the lowest content was a CWR (*L. dregeana*) and the one with the highest value was a red commercial variety (‘Red Sails’). A similar tendency was observed for the DI treatment the same year, with *L. dregeana* (21.23 mg 100 g^−1^ DW) and ‘Red Sails’ (356.90 mg 100 g^−1^ DW) again showing the lowest and the highest content in total anthocyanins, respectively. The differences were very significant (*p* = 0.003) and highly significant (*p* = 0.0005) in C and DI, respectively.

In 2021/2022, the values of the mean total anthocyanin content in leaves varied between 3.01 and 31.67 mg 100 g^−1^ DW in ‘Lollo Rosso’ and ‘Romired’, respectively, under C conditions, and between 4.32 and 35.57 mg 100 g^−1^ DW again in ‘Lollo Rosso’ and ‘Romired’, respectively, under DI conditions (**Table 2**; **Figure 2B**). Again, the differences were very significant (*p* = 0.001) and highly significant (*p* = 0.0003) in C and DI, respectively. As observed in the case of TAA content, the tendencies were comparable between treatments and years.

Interestingly, quite the opposite to what happened with TAA, in C conditions, the total anthocyanin content was higher in the stem than in the leaf in the wild species *L. dregeana* in both years (**Table 2**; **Figure 2B**), in total agreement with previous observations (Medina-Lozano et al., 2021). However, the situation was reversed under drought stress (DI); that is, leaves had more total anthocyanins than stems (in the same plants), as we will discuss in more detail below. The same three specific anthocyanins, cyanidin 3-*O*-(6’-*O*-malonylglucoside), cyanidin 3-(6’’-acetylglucoside), and peonidin 3-*O*-glucoside, found previously in a bigger set of accessions, including the ones studied here (Medina-Lozano et al., 2021), were also identified in this occasion (**Supplementary Tables S2**, **S3**).

As described before in another bushy lettuce wild relative, *L. squarrosa* (Medina-Lozano et al., 2021), in this work, the cyanidin 3-(6’’-acetylglucoside) was present only in the stems but not in the leaves of *L. dregeana* (**Supplementary Table S2**), under both C and DI conditions in 2020/2021 [cyanidin 3-(6’’-acetylglucoside) was not detected at all in 2021/2022 probably due to the low levels of total anthocyanins]. Cyanidin 3-(6’’-acetylglucoside) was not found in any of the cultivated varieties included in the previous study, though now we have detected it in two red-leaf commercial varieties, ‘Lollo Rosso’ and ‘Romired’ in 2020/2021 (**Supplementary Table S2**), probably due to the high quantities of total anthocyanins accumulated that year, as other authors have also reported it in some other red lettuce varieties (Wu & Prior, 2005; Viacava et al., 2017). Conversely, peonidin 3-*O*-glucoside, which was only observed in cultivated varieties in that same previous study, has also been detected here in the wild species *L. homblei* (**Supplementary Tables S2**; **S3**), but exclusively under DI treatment, as previously described.

The selected accessions covered all the spectrum in terms of anthocyanin content, from those with an absolute lack of them (the green commercial varieties ‘Dolomiti G12’ and ‘Winter Crop’) used to validate the method, or with a low content (i.e., the CWR *L. homblei*), to those with a high content (the red commercial varieties ‘Red Sails’ and ‘Romired’), with some accessions containing a medium amount in between (the red commercial variety ‘Lollo Rosso’ and the CWR *L. dregeana*), according to the groups described by Medina-Lozano et al. (2021).

In the case of anthocyanins, the response to drought stress was also very robust across the 2 years in which the two irrigation regimes (C *vs*. DI) were tested (**Table 2**; **Figure 2B**). The water stress conditions resulted in an increase in the total anthocyanin content in the leaf samples from all the accessions, both red-leaf commercial varieties and CWR in both years (as expected, anthocyanins could not be detected in the green-leaf varieties). Interestingly, the increase in total anthocyanin content in response to drought stress was significant and very significant in the case of the CWR *L. homblei* in 2020/2021 (*p* = 0.048) and 2021/2022 (*p* = 0.002), respectively (**Table 3**; **Figure 2B**).

The only case in which the total anthocyanin quantity was lower in plants under DI regime was in the stem tissue of the wild accession *L. dregeana* (**Table 2**; **Figure 2B**). Again, these results were reproducible across the 2 years. As mentioned before, stems contained more anthocyanins than leaves under C conditions, and it was exactly the opposite under DI conditions, where leaves had more anthocyanins than stems, and the changes in anthocyanins in the two tissues in response to drought stress were in inverse directions, increasing in leaves and decreasing in stems (**Table 2**; **Figure 2B**) in the same plant and for the three biological replicates (data not shown).

For the leaf samples, in all cases (all accessions and both years), the quantity of the major anthocyanin, cyanidin 3-*O*-(6′-*O*-malonylglucoside), increased as a consequence of water deprivation (DI). Only in the case of the stem samples did its quantity decrease, as expected because there was a drop in total anthocyanin content (**Supplementary Table S2**, **S3**).

## 4 Discussion

### 4.1 Antioxidant compound levels and variations under drought stress

#### 4.1.1 Total vitamin C

The results regarding TAA content in leaves under C conditions are in the same order of magnitude and in accordance with those obtained in a previous study in which these same accessions, among many others, were characterized for their quantity of vitamin C, which revealed that, generally, the *Lactuca* wild species had a higher content of TAA than the cultivated lettuces (Medina-Lozano et al., 2021). Something similar was observed by van Treuren et al. (2018) when comparing the AA content in lettuce varieties and some of their wild relatives.

AA and DHAA are easily interconvertible, though AA is the form with the highest antioxidant potential. That could explain why most cases in which DHAA was more abundant happened under C conditions, whereas under DI (drought stress), AA was predominant. Moreover, DHAA content was found higher than AA only in commercial varieties; that is, the lettuce wild relatives were always richer in the compound with more antioxidant capacity, AA (**Supplementary Tables S2**, **S3**).

The consistent detrimental effect of the water stress on the TAA content in our study totally agrees with what has been recently found in several leafy vegetables (Park et al., 2023) and in some cultivated lettuces (Ćavar Zeljković et al., 2023), in which drought also caused a decrease in their vitamin C amounts.

#### 4.1.2 Total anthocyanins

In contrast to what was obtained for vitamin C and in total agreement with our previous work (Medina-Lozano et al., 2021), the leaves from commercial lettuce varieties contained more total anthocyanins on average than the wild species assessed under C conditions.

Out of the three different anthocyanins found in this work and in a previous study also from our group (Medina-Lozano et al., 2021), cyanidin 3-*O*-(6’-*O*-malonylglucoside) was always present, and it was the most abundant one, as reported also by other authors (Wu and Prior, 2005; Mulabagal et al., 2010; Becker et al., 2014) whereas cyanidin 3-(6’’-acetylglucoside) was the rarest. However, some differences have been observed. For instance, ‘Lollo Rosso’ contained only cyanidin 3-*O*-(6’-*O*-malonylglucoside) in our previous study and in the second year of this study, while in the first year, it contained all three. Similarly, ‘Romired’ had only cyanidin 3-*O*-(6’-*O*-malonylglucoside) and peonidin 3-*O*-glucoside when it was previously characterized (Medina-Lozano et al., 2021) and in the second year of this work, while in the first year, the three anthocyanins were identified [though cyanidin 3-(6’’-acetylglucoside) was present only in the samples under DI treatment]. Similar cases are also observed in the CWR assessed. In general, when there was a high quantity of total anthocyanins, as it happened in 2020/2021, all three of them were present whereas when it was low, the less abundant ones could either not be detected or not be produced. Thus, what can be deduced from this is that some accessions have the genes to synthesize all those anthocyanins even if they are not produced all the time or even in all the parts of the plant. Actually, there were cases in which one of the minor anthocyanins was only synthesized when the plant was under drought stress, DI in our experiments [i.e., peonidin 3-*O*-glucoside in *L. homblei* in both years, and cyanidin 3-(6’’-acetylglucoside) in ‘Romired’ in 2020/2021; presumably, it would have been detected also in 2021/2022 if the total anthocyanin amount would have been higher] (**Supplementary Tables S2**, **S3**). That was actually observed in all the biological repeats (data not shown). These observations, together with the rise in the total amount of anthocyanins in all the tested accessions in response to the drought stress, point out a putative role of these compounds in the mechanisms deployed by the plants to tolerate these adverse conditions.

In most cases, only considering leaf tissue and C conditions, red commercial varieties had a higher content of total anthocyanins than the wild species, in contrast to what happened for vitamin C (**Table 2**). Both observations totally agree with results found by our group previously (Medina-Lozano et al., 2021). In the case of anthocyanins, that was expectable as red-leaf varieties have been bred to enhance their anthocyanic color because it has a positive effect on their market price (Missio et al., 2018).

The significant increase of anthocyanins in *L. homblei* under water stress observed in both years points out to a potential source of drought tolerance in this wild species that could be easily introduced in the cultivated lettuce as they both belong to the same genus *Lactuca*. It is actually common for plants to synthesize different compounds to actively fight against adverse conditions, for instance, sugars, antifreeze proteins, or heat shock proteins in response to low temperatures, frost, and high temperatures, respectively, among others (Catalá et al., 2012). These results are in total agreement with our hypothesis of anthocyanins playing a role in the response of lettuce-related species to drought stress. In line with this, a recent study in vine has demonstrated the induction of *WRKY40*, a transcription factor that regulates anthocyanin biosynthesis, in the berry skin when the plants were subjected to severe water stress (Carvalho et al., 2023).

Under drought stress, the situation is the opposite in terms of anthocyanin content in the two tissues studied. That is, under C conditions, the stems had a higher amount of anthocyanins than the leaves, whereas under drought stress, the leaves contained more anthocyanins than the stems. These results could suggest a translocation of these antioxidant compounds as a first quick mechanism to cope with a severe lack of water in a tissue with a high susceptibility to water deprivation such as the leaf (from less to more susceptible tissues to dehydration). In this way, anthocyanins would be located where they are most needed when plant survival is under threat. It seems that there are routes for the transport of anthocyanins at subcellular, cellular, tissue, and organ levels (Zhao & Dixon, 2010 and references herein) though their regulation, specially under stress, has not been properly studied yet. This putative movement of phytochemicals in response to water stress was not observed in the case of TAA, which makes sense as its quantity decreases under DI, which rules out TAA having a role in the drought tolerance response.

The rise in anthocyanins as a consequence of the lack of irrigation reported in this study is in line with the results reported by Ćavar Zeljković et al., (2023) that observed an increase in some phenolic compounds (free phenolic acids and flavonoids) in response to drought in some cultivated lettuces, though the authors did not include anthocyanins among the flavonoids quantified.

The inverted tendencies shown by TAA and total anthocyanin contents in response to the deficit irrigation (**Figure 1**; **Table 2**) lined up with our previous observations, that is, the existence of a negative correlation between the content of both types of compounds (Medina-Lozano et al., 2021).

## 5 Conclusions

Water deprivation caused a drop in vitamin C content in all accessions within all groups and in both tissues—leaf and stem—analyzed. The explanation to this could be that, in a critical moment in which the survival is under threat, the plant could shut down all the processes that are not directly involved in survival and tolerance to drought stress, in this case. Another non-exclusive reason could be that the rise in anthocyanin biosynthesis could cause a reduction in other processes of the plant that compete directly for the same resources. Quite the opposite to what happened to vitamin C, the anthocyanin content increased under water stress in the leaf samples from all accessions within all groups (it only decreased in the stem samples). According to the hypothesis just revealed, it is reasonable to think that anthocyanins could play a role in the mechanisms deployed by the plant to cope with the drought stress though this needs to be verified in future experiments. If so, these would be promising plant materials for breeding more drought-tolerant lettuces that are also rich in some compounds that provide health benefits. Interestingly, a significant enrichment in anthocyanins as a response to the drought stress was observed in one of the wild relatives assayed (*L. homblei*). It is well known that CWR are a source of resistance in the case of biotic stresses. Similarly, it is expected to find higher levels of tolerance to abiotic stresses among the CWR as they are exposed to adverse climatic conditions usually mitigated for the crops by any agricultural system.

The challenge ahead is to decipher the role of anthocyanins in drought tolerance to engineer them and improve the lettuce resilience and, as a positive side effect, its benefits to human health. If so, a disadvantageous situation like drought could be turned into something beneficial in terms of plant fitness and human nutrition. Furthermore, it would also be possible to reduce the volume of irrigation water without paying any production penalty, which has undeniable environmental and economic upsides.

## Data availability statement

The original contributions presented in the study are included in the article/**Supplementary Material**. Further inquiries can be directed to the corresponding author.

## Author contributions

IML: Data curation, Formal analysis, Investigation, Methodology, Writing – review & editing. JRB: Data curation, Investigation, Methodology, Writing – review & editing. AD: Data curation, Investigation, Methodology, Writing – review & editing, Conceptualization, Formal analysis, Funding acquisition, Project administration, Supervision, Validation, Writing – original draft.
